# Hospital and Institutional News

**Published:** 1912-06-08

**Authors:** 


### HOSPITAL AND INSTITUTIONAL NEWS.

### ST. BARTHOLOMEW'S HOSPITAL.

The Lord Mayor, Sir Thomas Crosby, M.D., gave a dinner to the Treasurer, Governors, staff, and supporters of St. Bartholomew's Hospital on the 5th instant. As the dinner will not be held until after we have gone to press we are unable to report the proceedings this week. It is nine years ago since the Lord Mayor of the day displayed similar hospitality to the London Hospital, when it was very sorely pressed for money, and so we believe commenced a new departure which the present Lord Mayor deserves recognition for having followed in the cause of St. Bartholomew's Hospital.

### A SIXTY-HOUR WEEK FOR MENTAL HOSPITAL WORKERS.

Dr. A. R. Urquhart, Superintendent of James Murray's Royal Asylum, Perth, in The Hospital, last week, deprecated Parliamentary interference with nursing questions in the Scottish mental hospitals. We agree with him, and hope that it may prove unnecessary. It is most desirable that amelioration in the conditions of service of those engaged in nursing the mentally afflicted should be effected by the local authorities and Lunacy Boards of England and Scotland without compulsion by Act of Parliament. If, however, the average number of hours worked per week be 84 or even near that figure, it is impossible, as we pointed out in a recent leading article, to say that the demand for a reduction is not justifiable. It is time that the local authorities took action. Dr. Urquhart dreads the introduction of "the factory system of hours of work" into mental hospitals. We share with him this dislike, but cannot see why he thinks it imminent. The 70 hours per week limit proposed by the Select Committee of the House of Commons can be worked without introducing a factory system of hours. In our opinion some reduction of the present average working hours would operate to the advantage of both attendant and patient.

### "PAYING PATIENTS" IN A POOR-LAW INFIRMARY.

Mr. G. T. W. Newsholme, chairman of the Sheffield Board of Guardians, is sponsor for a scheme, involving an expenditure of between £15,000 and £20,000, by which it has been decided to extend the Poor-Law Infirmary. The children's block will receive 100 additional beds; 24 further mental cases will be provided for; the maternity block will be improved; and a three-storeyed block for 96 beds will be added. Mr. Newsholme remarked at the decisive meeting that in the past five years the number of patients had almost doubled, and, in fact, nearly double the number of children provided for were being housed at the present time. The new block, he anticipated, would be required, though probably not ready, by next winter. The Hospital's Extensions Sub-Committee recommends the various appointments, including, as we note elsewhere, a second assistant dispenser. These appointments were severely criticised by Father P. Hickey, who affirmed that the present staff was underworked, and Councillor H. Bolton made the surprising suggestion that "a middle-course hospital" should be decided upon in place of the extensions, where "patients could pay a small sum in respect of the treatment they received." He suggested that the Winter Street Hospital "might be suitable for the purpose." After Father Hickey had expressed the opinion that Councillor Bolton's suggestion would "meet the situation in every way," Mr. Newsholme explained from the chair that the Guardians had nothing to do with paying patients. It was left for the vice-chairman, Mr. J. W. Flint, to point out that the City Council and not the Poor Law could provide for non-destitute persons. That it should need pointing out at all at a committee meeting of progressive Guardians is astounding enough.

### EUGENICS IN PRACTICAL FORM.

From across the Atlantic comes the news that the Bishop of the Protestant Episcopal Cathedral of Chicago has announced that no couples will in future be married in the cathedral unless each of them produces a certificate of health from a reputable physician, to the effect that they are normal physically and mentally and have neither an incurable nor a communicable disease. We forbear to dwell upon the obvious difficulties in which the phrase "reputable physician" may involve the Bishop. Nor can we here discuss the wisdom or expediency of the step which he has thus taken. But as an example of the way in which educated people are awakening to the problems of race improvement to which Galton devoted his life it is at least interesting. The high position of the cleric who has taken this first step will at least ensure a widespread discussion and ventilation of the whole subject of the medical regulation of the marriage contract.

### THE PATHOLOGICAL BLOCK OPENED AT CARDIFF.

The formal opening of the Pathological Department at King Edward VII. Hospital, Cardiff, took place last Saturday. Mr. W. James Thomas, who has contributed in all some £18,000 to the hospital's funds, was introduced by the Lord Mayor, Alderman J. W. Courtis, at a preliminary meeting in the Llanbradach ward. Mr. Thomas described the opening of the new department as a step in the evolution of the hospital which would affiliate it still more closely to University College, Cardiff. All the material is at hand, he said, for a great Welsh medical school, but its creation cannot be accomplished till the statutory number of beds are opened; that is why the remaining 50 beds in the new wing should be opened at once. Sir Isambard Owen, Vice-Chancellor of Bristol University, referred to his previous work in connection with the establishment of a medical school in Cardiff, a work which began in 1889. Through Mr. Thomas's liberality they could teach their students medicine and surgery in addition to the zoology, physics, botany, and chemistry which were taught at present. Pathology, he proceeded, had already tracked malaria and yellow fever, while by it Malta fever should very soon become a thing of the past. To pathology tuberculosis also would one day yield. That is why he was glad that something was being done for pathology in Cardiff. Dr. H. A. Scholberg and Prof. Emrys-Roberts, the honorary pathologists, proposed and seconded a vote of thanks. After speeches had been made by Principal Griffiths and Dr. C. T. Vatchell, Lieut.-Colonel Bruce Vaughan handed the key of the building to Mr. Thomas, saying: "It is a great pleasure for me to state here publicly that it is by your noble generosity that not only is this building completed, but that all the beds in the new wing will very soon be opened. I cannot but associate with you in this great work the names of Lord and Lady Bute, Lord and Lady Aberdare, a 'Glamorganshire Owner of Property,' Mrs. John Nixon, and Lord Merthyr." In a word, the occasion notified a great step towards the proper housing of the medical school.

### THE RESEARCH DEPARTMENT IN DETAIL.

The new Pathological Department at King Edward VII. Hospital, Cardiff, has been built by Mr. Bond from the designs of the hospital's hon. architect, Colonel E. M. Bruce Vaughan. It consists of a basement, a ground floor, and two upper storeys. Starting from the former we find an ice-making plant, an electrically-driven motor, and a refrigerator, two store-rooms, one for the hospital and one for University College, a sterilising room, and apparatus for the preparation of the media in bacterial work. There is also a dark-room and a room for photo-micography. The ground floor contains the post-mortem room, the mortuary, and mortuary chapel, which has a mosaic pavement, tiled walls, a painted ceiling, and carved woodwork. Dr. Scholberg's chemical laboratory, with compressed-air and steam apparatus and electric motors, is, together with cloak-rooms, also on the ground floor. The director's office, Prof. Emrys-Roberts' room, with his own experimental apparatus, the demonstration room, with accommodation for sixteen students and the fittings of which were designed by the Professor, a clinical laboratory, two research laboratories, and an incubation room comprise the first floor. The museum, which occupies the second floor, has a fine window extending its full length, and it measures 38 feet by 36 feet, and on this floor also are a class-room and a students' reading-room. All the floors are connected by lifts.

### MR. JOHN BURNS ON INFANTILE MORTALITY.

Under the patronage of the King and Queen a National Association for the Prevention of Infantile Mortality and the Promotion of the Welfare of Children under School Age has been formed. Mr. John Burns, M.P., presided on Tuesday at a meeting which had this happy result, and in supporting the project he said that their two previous conferences had done a great deal to promote their object. When they began their work the infant mortality rate stood at the relatively high figure of 145 per 1,000. In six years the number had diminished to 105 per 1,000. The improved conditions had been brought about first by the doctor, secondly by the nurse and the midwife, and then by benevolent people. Last year they struck 1,144 milk shops off the register because milk was being sold in unsuitable premises. He wished that he could do the same for public-houses. If he could do it by an Order of the Local Government Board, the Order would be issued on the following day. Sir Thomas Barlow seconded the resolution, which was carried unanimously.

### MR. PEASE, M.P., ON THE SCHOOL CLINIC.

"Pending the establishment of a more complete system of school clinics," states the Board of Education, "it is inevitable that the hospitals should continue to be utilised for work which it may not be desirable for them to undertake permanently, and in these circumstances the Board are prepared to give favourable consideration to the proposed arrangement with the Metropolitan Hospital" for the medical treatment of elementary school children, for which the London County Council has sought its sanction. The reason why the Board favours school clinics is given in another passage from their reply, which we also quote: "It is clear that the control exercised by a local education authority over the treatment of children at a hospital, which exists primarily for other purposes, cannot be so complete as the control exercised in the case of a school clinic." The deputation to Mr. Pease, which was introduced by Sir Victor Horsley, is evidently bearing fruit.

### PROFESSOR PEARSON ON SANATORIA.

Fortunately for Professor Karl Pearson, society no longer prescribes an overdose of hyoscyamus for those who spend their time upsetting its most cherished preconceptions in the Socratic spirit of unbiased inquiry. Fortunately, too, the splendid endowment of his forerunner provides him with the means to carry on his researches and to jog us all rudely out of our self-complacency. And what a mercilessly calm critic he can be! In a lecture delivered three months ago, now republished (Dulau and Co., 1s. net), he applies his methods of mathematical analysis to the tuberculosis problem, and pleads earnestly for a revision of the proposal to spend a million and a-half on sanatoria. We are not concerned for the moment to judge whether or not he succeeds in establishing the proposition that, broadly speaking, sanatoria have failed as a remedy for tuberculosis, but we are certainly at one with him in his protest against the illogical and unscientific way in which only too many public-health officers deal with statistics. Professor Pearson confesses himself a socialist. He demands a higher standard of scientific training in the medical advisers of Government departments and some cessation of charlatanism in politics. The educated, he says, will never form a voting majority, but they can, if they will make the effort, be dominant by the written word.

### HULL ROYAL INFIRMARY'S INCURABLES HOME.

The new Home for Incurables, under the management of the Hull Royal Infirmary and known as the John Symon's Home for Incurables, has been opened, and patients are now being admitted. There is accommodation for ten patients, and it is proposed to take both free and part-paying cases as the funds will allow. This Home, it should be recalled, was founded by the bequest of the late Alderman John Symon, a noteworthy Hull citizen, whose eccentricity and generosity endeared him to many.

### THE NEW CANCER HOSPITAL AT GLASGOW.

The Royal Cancer Hospital, Glasgow, which Princess Louise opened last week, has been reconstructed and enlarged at a cost of £27,000. Dr. D. J. Mackintosh, M.V.O., has superintended the alterations, which have been in progress for a considerable time. The hospital has accommodation for 40 patients, and a research department. The building consists of three storeys, and the large wards are on the ground floor, while provision has been made for several single wards for isolation patients, and the convalescents have a ward to themselves. The large wards are 40 feet long, 26 feet wide, and 13½ feet in height. The beds are of the "balcony" pattern. The floors, which are finished with polished pitch pine, are laid on fire-proof concrete, and noise is obviated by a layer of felt. The walls are partly tiled, the annexes are in towers, and the ward unit comprises in addition a douche room, water sterilisers, ward kitchen, duty room, and linen press. The corridor floors and those of the annexes are of terrazzo, while care has been taken, by projecting the north ward beyond the south, to secure direct south light to the former. Each has a balcony. The research department occupies most of the top flat. The basement contains the kitchen and store-rooms, while behind the main building are the laundry, destructor, and steam disinfector. Messrs. James M. Monro and Son are the architects, and it is satisfactory to note that the building has been opened and equipped free from debt. Princess Louise was received at the new nurses' home by Mr. Robert Gourlay, LL.D., president of the hospital. Sir George Beatson, K.C.B., and the Lord Provost were among the speakers.

### A NEW SECRETARY AT HULL.

A new secretary has been appointed, in the person of Mr. C. W. Stanford, to the Victoria Children's Hospital, thus filling the vacancy caused by the death of Mr. W. D. Theaker. Mr. Stanford has acted for some time for the late secretary, and is therefore acquainted with the work of the Children's Hospital, but in his new capacity the sphere of his usefulness will be enlarged, and we wish him every success.

### RATION RATES FOR INFIRMARY OFFICERS.

The vexed question of payment for rations to officers when on their annual leave of absence was discussed by the Wandsworth Board of Guardians at their meeting on Thursday last. Several male officers at St. James' Infirmary had applied to the committee for the allowance, but the latter recommended the Board to take no action. The Guardians, however, by a majority of one, referred back the committee's report for further consideration. It was pointed out that the Local Government Board had refused to sanction the allowance when an application was made to them in 1900. The salaries were arrived at when the appointments were made on the basis that the officers would have an annual leave, and this was taken into account when the repayments from the Metropolitan Common Poor-Law Fund were made. The Board, however, thought that in the intervening twelve years some alteration had taken place at Whitehall, and that if another appeal were made to the Local Government Board the payments for rations when on leave might be sanctioned.

### ENGLISH ARCHITECTS AND WELSH SANATORIA.

The Executive of the Welsh National Memorial invite architects to submit designs for two sanatoria; one in North Wales to contain 150 beds and one in South Wales to accommodate 250. The sketch designs will be treated as confidential, and the Executive will pay premiums to several architects, who will be invited further to elaborate their designs. Particulars may be obtained from the Secretary to the National Memorial, Newtown, Montgomeryshire. It will be noted with satisfaction and amusement that the present exigencies of party politics have not entered the sphere of public health in Wales, nor confined the competition to Welsh, to the exclusion of "foreign," competitors.

### A SURGEON AND ATHLETE.

Mr. R. B. Etherington-Smith, M.B., F.R.C.S., has been elected assistant surgeon at St. Bartholomew's Hospital in place of Mr. McAdam Eccles, promoted to the full staff. Mr. Etherington-Smith was famous thirteen or fourteen years ago as a Cambridge University and Leander boatingman, and was one of the finest heavy-weight oarsmen that has rowed against Oxford. He has since then frequently coached Cambridge crews; and has had a distinguished career at St. Bartholomew's, where he is warden of the college. He has lately been surgical registrar at St. Bartholomew's, and is also assistant surgeon to the West London Hospital. The appointment will be immensely popular with students and staff alike; and is an admirable one in every way. Mr. Etherington-Smith is not only an athlete and a surgeon, but also a man of character whose influence is wide and is exercised for good.

### A SANATORIA SCHEME FOR ESSEX.

A scheme for dealing with consumption under the Insurance Act was outlined at Chelmsford on Tuesday by Dr. J. C. Thresh, the County Medical Officer, before a conference of local authorities convened by the Mayor. Dr. Thresh said it would be necessary for the county to provide 200 beds in a central sanatorium; and for dispensary purposes he proposed to divide the county into nine centres, each with subsidiary centres. For the Chelmsford area, in which it was proposed to combine the boroughs of Chelmsford, Maldon, and various urban and rural districts, he estimated the total cost of the scheme would be £4,600 per annum; of this £3,070 would have to be raised by rate. The conference approved the principle of the provision of the county sanatorium by the county council, but maintained that the dispensaries should be provided and maintained by the local areas. A similar conference is to be held in each of the nine areas suggested by Dr. Thresh, who will afterwards lay their views before the county council.

### STATISTICAL PUZZLES.

The Cardiff Mental Hospital has often claimed a high recovery-rate, and the latest annual report states that " the recovery-rate, which during 1910 was considerably above the average, reached an extraordinary figure for 1911, viz., 60 per cent. . . Of those who left recovered 64 per cent. did so in not more than eight months after reception." The maintenance charge has been raised from 12s. 3d. per head per week to 12s. 6½d. in consequence, says the report, of concessions. Advantage of these statements was taken by Alderman F. J. Beaven to affirm that a number of the discharged patients were coming back. To this Dr. Goodall, the medical superintendent, retorted that the returns were largely due to the miserable conditions in which some of the ex-patients lived after leaving the institution. Alderman Beaven thereupon asked how it was that the maintenance charge had increased. Dr. Goodall explained that now the hospital had fifty-four fewer patients than when it was opened, and that this smaller number decreased the total cost, though the cost per head was higher—which is a good and simple example of the skilled handling which statistics require.

### ISOLATION ACCOMMODATION IN FEVER HOSPITALS.

As usual report after report comes to hand showing the serious lack of isolation accommodation in the form of separate wards which is so prevalent in provincial fever hospitals. Another example similar to those we have already noticed is given in the report of Dr. James McGregor, Medical Superintendent of the Milton Fever Hospital, Portsmouth. This hospital, which was built for 122 beds, now contains, he states, 189. Dr. McGregor complains of the difficulty of classifying scarlet fever cases. In the present diphtheria ward divisions exist for isolating cases of mixed infection, and Dr. McGregor properly asks that two similar wards should be built, which would not only increase the accommodation by 72, but by providing more adequate isolation units it might make it possible to admit " bad cases of measles, whooping cough, or similar diseases in times of epidemic from very poor houses, the mortality being very high in children treated at home." It is to be hoped that the medical superintendent will receive more support than has been shown to his proposals hitherto, and that the Guardians who have objected to admitting an infectious patient to the infirmary may recall the Corporation to its duty in providing sufficient accommodation in the infectious diseases hospital.

### THE MURAL DECORATION OF MIDDLESEX HOSPITAL.

The Exhibition of Designs for Mural Painting, and for the Decoration of Institutions, which we announced on April 20, opened last Monday at Crosby Hall, Cheyne Walk, Chelsea. It has a special interest for hospital workers from the fact that the designs include those sent in for the competition which, thanks to Mr. Edmund Davis, has been inaugurated for the decoration of the vestibule of the Middlesex Hospital that he has generously undertaken to rebuild. Five competitors have sent in panels, and if this number is strikingly small that is because the competition has not been advertised sufficiently. There seems little doubt, indeed, that had the competition, for which Mr. Davis has offered a prize of £100 per panel, been more widely known there would have been a far larger selection from which to choose. One artist, for instance, who knows a considerable number of the craft, was heard to remark last Saturday that the occasion of the private view was the first day on which news of the competition had reached him. If this be the case with one there can be little doubt that his unlucky ignorance has been shared by others. Remembering the probability of this, we will not criticise the designs too closely. All the competitors, however, seem to have failed adequately to grasp two facts. The first is that for mural decorations of this class very broad treatment is essential, so far as it can be made compatible with decorative effect; and, secondly, that though the vestibule of a hospital is not the out-patients' hall, yet the decorations are like poster work in this, that presumably they are intended to appeal to ignorant people. There must be no difficulty for their public in grasping at once the intention of the design. The problem is to combine decorative with illustrative treatment; in short, to produce the best traditions of first-rate poster work. The moral for Mr. Davis, if we may venture to say so, is that it would be worth while to extend the period before which designs must be sent in to, say, a further three months. If this were done, and advantage taken of it to advertise the competition among artists generally, we have little doubt that the designs sent in would be not only better, but more numerous.

### FROM A SURGERY WINDOW.

The opposition of the medical profession to the Insurance Act is probably as strong in Manchester as anywhere, and the window of one surgery in Salford contains the typical notice that " Medical aid will not be given here under any terms of the National Insurance Act." It has further been decided, we learn, that all information requested by the civic authorities shall be uniformly disregarded, "and if necessary strenuously resisted." No doubt Sir James Barr's activity has not been lost upon Northern members of the medical profession. The North-Country newspapers, too, are bubbling over with medical correspondence on the subject of the Act, and most of the writers, we are glad to note, have had the good sense not to write anonymously.

### INSURANCE QUESTIONS AND HOME RULE.

SIR DONALD MACALISTER, President of the General Medical Council, presided at the 95th session on Tuesday. The following were appointed members of the Committee to deal with questions under the Insurance Act: Sir Donald MacAlister, Dr. Mackay, Dr. Norman Moore, Dr. Langley Browne, Sir David McVail, Sir Charles Ball, Dr. Saundby, Dr. Latimer, Dr. Norman Walker, Dr. Kidd, Sir F. Champneys, Mr. Hodsdon, and Sir Arthur Chance. Thereupon Sir Charles Ball moved that "steps should be taken to procure the insertion in the Government of Ireland Bill of provisions reserving to the Imperial Parliament the control of legislation relating to the Medical and Dentists Acts." He added that "it would be within the power of the Irish Parliament to make or amend laws relating to medical practice; and it is possible that, if such were done, the standard would not be lowered." On the other hand, he believed that there would be an agitation for a lowering of the standard to an extent that would not be permitted in this country.

### A TUBERCULOSIS EXHIBITION FOR BIRMINGHAM.

DURING July a Tuberculosis Exhibition will be held in Birmingham, when models, diagrams, and pictures will be shown illustrating the causes of consumption and the best methods of preventing it. Lectures by medical men will be given, and each day, during the fortnight over which the exhibition will extend, 450 children from elementary schools will be invited. It is much hoped also that men and women of the working classes will attend, and to induce them to do so admission will be free. To secure the financial solvency of the exhibition a guarantee fund of £450 is being raised. In this connection we may also note that at the sessional meeting of the Royal Sanitary Institute, to be held at Torquay on July 5, there will be a discussion on "Tuberculosis and Sanatorium Benefits under the Insurance Act," which Dr. George Adkins, Medical Officer of Health for Devon, will open.

### RESIGNATION OF A DISPENSER.

MR. W. B. FITCH, dispenser to St. Bartholomew's Hospital, Rochester, has sent in his resignation. At the end of March Mr. Fitch wrote to us asking whether "it is customary or desirable that the matron of a hospital should have the keys of the dispensary," and protesting against her having them. Our answer was that good will among hospital staffs was generally sufficient to decide the point. Mr. Fitch, however, has not let the matter rest. He wrote to the professional press on the point, and has since published the comments of THE HOSPITAL and other technical journals in the local paper. No doubt this action has not tended to smooth over this unfortunate squabble, but the principle for which he is contending solves itself in large institutions, and should not be allowed to create discord in smaller hospitals where the different departments are inevitably less structurally and otherwise defined.

### A WELL-KNOWN IRISH SURGEON.

AN interesting memoir of the late Sir William Thornley Stoker, a well-known Irish surgeon, appeared in the *Times* on Monday. The public is reminded that three of his brothers were the late Mr. Bram Stoker, Sir Henry Irving's coadjutor; Colonel Richard Stoker, of the Indian Medical Service; and Dr. George Stoker, C.M.G., who initiated and organised the civil hospitals in South Africa and served with the Irish Hospital during the war. Sir Thornley's younger sister is the widow of Sir William Thomson, of Dublin, who also served in South Africa as Surgeon-in-Chief of the Irish Hospital. Sir Thornley studied medicine in Queen's College, Galway. He graduated in 1866, and for a number of years he prepared students for examination. In private practice he soon became known as a surgeon. He was a former surgical Fellow of the Royal University, president of the Royal College of Surgeons in Ireland, and president of the Royal Academy of Medicine. The honour of knighthood was conferred upon him in 1895, and he was made a baronet in 1911. He found time to write numerous papers on surgical subjects. He took also a deep interest in music, the drama, and in literature. He was also connected with many Dublin charities. The baronetcy becomes extinct.

### A MEMORIAL TO A HOSPITAL SECRETARY.

MRS. H. BONNETT, of Cambridge, has offered to build, equip, and fully endow a clinical laboratory for the benefit of Addenbrooke's Hospital to perpetuate the memory of her son the late Mr. John Bonnett, M.A., who was for many years secretary and legal adviser to the hospital, and one of its most ardent advocates and supporters. Seldom, if ever, has such a generous or more valuable benefaction fallen to the lot of Addenbrooke's Hospital. In the past the clinical work of Addenbrooke's Hospital has for the most part been carried out at the medical schools attached to the University, but to be able in the future to have this important work carried out in the premises will prove a tremendous boon to the physicians and patients. This generous benefaction has come at an opportune time, seeing that the governors of the hospital are about to decide on the plans for the much-needed new wards and offices for children, new out-patient department, new boiler house and boilers, additional accommodation for nurses, and an electric lift, etc., and it is to be hoped that such a gift may serve as a stimulus to others interested in the institution forthwith to provide the £6,000 or £7,000 still required to enable the governors to have the whole scheme for extension completed.

### FROM DISPENSER TO PHARMACIST AT SHEFFIELD.

The Board of Guardians at Sheffield have decided to give the title of "pharmacist and dispenser" to the Union's dispenser. To hospital authorities this apparent juggling with words really marks a change of considerable importance, because, whilst a dispenser may have a certificate of no great value, a pharmacist has his title by virtue of the diploma of the Pharmaceutical Society, which certifies efficient training. Mr. Herbert Antcliffe, M.P.S., is the dispenser whom the Board has been pleased to honour. He holds one of the most important Poor-Law dispenserships in the country, and is, in addition, secretary of the Sheffield Pharmaceutical and Chemical Society. We notice also that, when deciding to appoint a second assistant dispenser, the Board stipulated that applicants shall be registered as pharmacists under the Pharmacy Acts.

### THE AMERICAN HOSPITAL IN PARIS.

Thanks to the passing of a Bill by the United States Senate the American Hospital in Paris has taken the first step towards gaining a charter. No opposition is expected from the House of Representatives. The Bill was drawn up by Mr. Henry Cachard, secretary of the hospital, which, visitors to Paris will remember, is situated at the corner of the Boulevard du Château and the Rue Chauveau, in Neuilly-sur-Seine. When passed into law the Bill will allow the hospital to receive bequests, grants, and other property, which will thus make its extension an easier matter than at present. Probably hospitals, as being wholly free from money-making, are the only institutions in Europe for which a special charter would be sanctioned by Congress. In any case, of course, the hospital is inspected by the French authorities, and those interested in securing the charter did not fail to point out that the French Hospital in London and that in New York are thus incorporated by the French Government. The hospital, which has been established only for a few years, has a fine garden, private rooms, and a free ward, and the passing of the Bill into law is expected to be followed by its extension.

### A LONDON HOSPITAL AND ROYAL FAVOURS.

The King's Levee taking place on Thursday last week, Sir Douglas Powell presided in place of Prince Alexander of Teck, who had to be at St. James's Palace, at the quarterly court of Governors of the Middlesex Hospital. Mr. Reginald Lucas made special allusions to the recent royal favours which this hospital has received, as instanced by the Queen's recent visit, and by the continued interest taken by Prince Alexander to which the special ward owes its existence. The redecoration of the entrance hall, discussed elsewhere in The Hospital, which Mr. Davis has undertaken, was also referred to, and Mr. Clare Melhado, the secretary, stated that the subscriptions for the past quarter had been practically maintained.

### THE LIVINGSTONE CENTENARY.

As we go to press the commemoration proceedings at Livingstone College, Leyton, are being held. Dr. C. F. Harford, the Principal, is being followed by the Rev. R. Wardlaw Thompson, who, as representing the London Missionary Society, under which Dr. Livingstone went forth as a missionary, commends the proposal to inaugurate a Livingstone Centenary Fund in connection with Livingstone College as part of the commemoration of the centenary of Dr. Livingstone's birth, which will take place in 1913. Sir Havelock Charles, G.C.V.O., M.D., Serjeant-Surgeon to King George V., and the medical attendant to the King and Queen during their recent visit to India, delivers an address. Anything which tends towards the essential reform of missionary work, which entails that every missionary should have some medical education, is to be welcomed. As everyone knows, the worst blunders in the mission field have been due to the workers having had no medical or scientific training.

### THE MEDICAL PROFESSION AND THE LORD MAYOR OF LONDON.

Attention has several times been called in these columns to the manifold energies of Sir Thomas Boor Crosby, M.D., Lord Mayor of London, and the first medical man to fill that proud position. It is therefore with pleasure that we notice that Councillor S. H. Curry, L.R.C.P., of the Battersea Borough Council, is endeavouring to promote a presentation and a dinner to Sir Thomas, to be subscribed by medical men doing public work, whether as members of Parliament, county, borough, rural or urban district councillors, or on boards of guardians. Councillor Curry appeals to all doctors holding any such positions to send him their names, as no directory exists which enables him to communicate directly with them on the subject. We think that Mr. Curry might well include also medical liverymen of the City Companies; and we wish him all success in his effort to recognise fittingly the great services which Lord Mayor Crosby has conferred upon the City and the country, and the reflected honour which his period of office has shed upon the medical profession.

### THIS WEEK'S DRUG MARKET.

Business in the Drug market is still very quiet pending a complete settlement of the strike, which, fortunately, seems in sight. There are few changes in value of any importance to report. The tendency in the Opium market is in a downward direction, as a fair crop is expected. Morphine still tends lower in value, and the price of apomorphine has been reduced. The price of saffron has advanced. Buchu leaves are dearer, and will probably continue to advance for some little time. Cardamoms are also dearer. Arnica flowers are scarce and dearer. Small crops of hyoscyamus and belladonna are expected as a result of the recent drought. Oil of cloves is again dearer. Gentian root is scarce and dear. Higher prices for rhubarb are expected.

